# Supplementary material for: Psilocybin during the postpartum period induces long-lasting adverse effects in both mothers and offspring
Source: Nat Commun. 2025 Sep 30;16:8630. doi: 10.1038/s41467-025-64371-5 (PMC12485072; doi:10.1038/s41467-025-64371-5)
Supplement: Supplementary file 1 — Supplementary Information [file 41467_2025_64371_MOESM1_ESM.pdf]

Psilocybin during the postpartum period induces long-lasting adverse effects in both mothers and offspring

Supplementary Fig. 1: Psilocybin is less potent in parous vs. non-parous female mice.

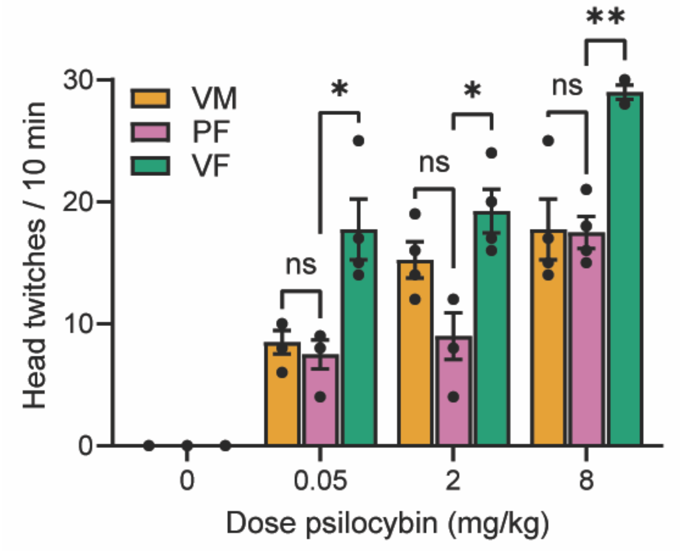

Number of head twitches in a 10 min period among virgin males (VM), postpartum females (PF), and virgin females (VF) following psilocybin injection at the given doses. Data are presented as mean ± SEM. Data were analyzed with a 2way ANOVA followed by planned comparisons between postpartum females and all other groups. *p < 0.05, ns = not significant

Exact P values are provided in the Source Data.

Supplementary Fig. 2: Maternal sucrose preference correlates with pup preference in stress-exposed dams.

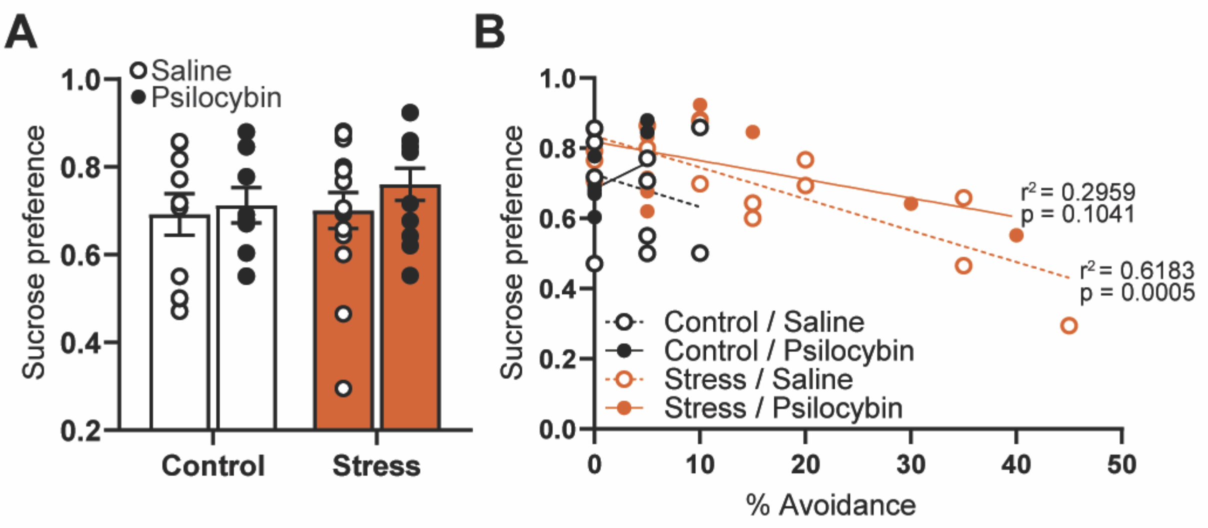

(**A**) Sucrose preference measured in dams on PPD 12, and (**B**) correlated with pup avoidance on PPD 8. Data in **A** are presented as mean ± SEM. Data in **B** are presented as individual sucrose preference values and pup avoidance values, and a line of best fit analyzed using a two-tailed Pearson correlation analysis. Goodness of fit lines for stressed animals with or without psilocybin treatment are labeled as r^2 on the graph. Exact P values are provided in the Source Data.

Supplementary Fig. 3: Psilocybin specifically produced an anxiolytic phenotype in virgin females.

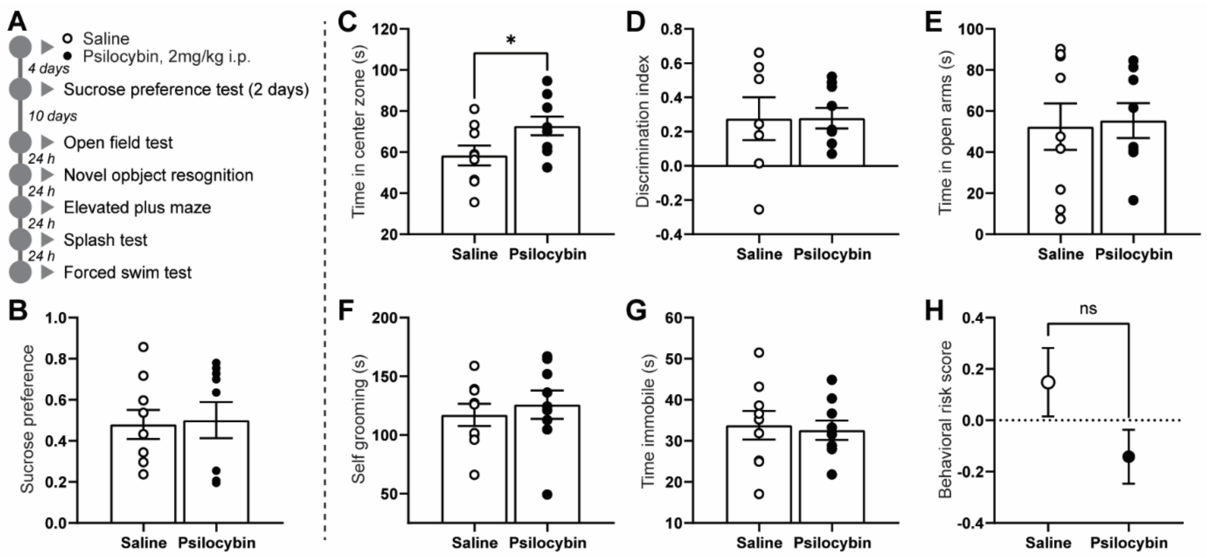

(**A**) Timeline of behavioral battery. Data illustrated in **B–G** represent primary end-points measured in the battery completed two weeks following psilocybin exposure. (**B**) Sucrose preference measured 6 days after psilocybin injection. (**C**) time spent in the center zone of the open field test. (**D**) discrimination index indicating the relative time spent investigating a novel object compared to a known object, $(t_{\text{novel object}} - t_{\text{familiar object}}) / (t_{\text{novel object}} + t_{\text{familiar object}})$. (**E**) time spent in the open arms of the elevated plus maze. (**F**) Time spent self-grooming in the splash test. (**G**) Time spent immobile in the forced swim test. (**H**) Integrative behavioral risk score, an integrative measure of global impairments in the test battery calculated by averaging z-normalized results from the primary end-points of each behavioral test for each animal, such that that greater the risk score, the greater the global impairment. Individual data in **B–G** are presented as aligned dot plots and as bar charts representing mean ± SEM. Data in **H** are presented as mean ± SEM. Statistical comparisons between experimental groups are made using a t-test. N = 9 mice/group. *p < 0.05, ns = not significant. Exact P values are provided in the Source Data.

Supplementary Fig. 4: Psilocybin acutely impairs maternal care.

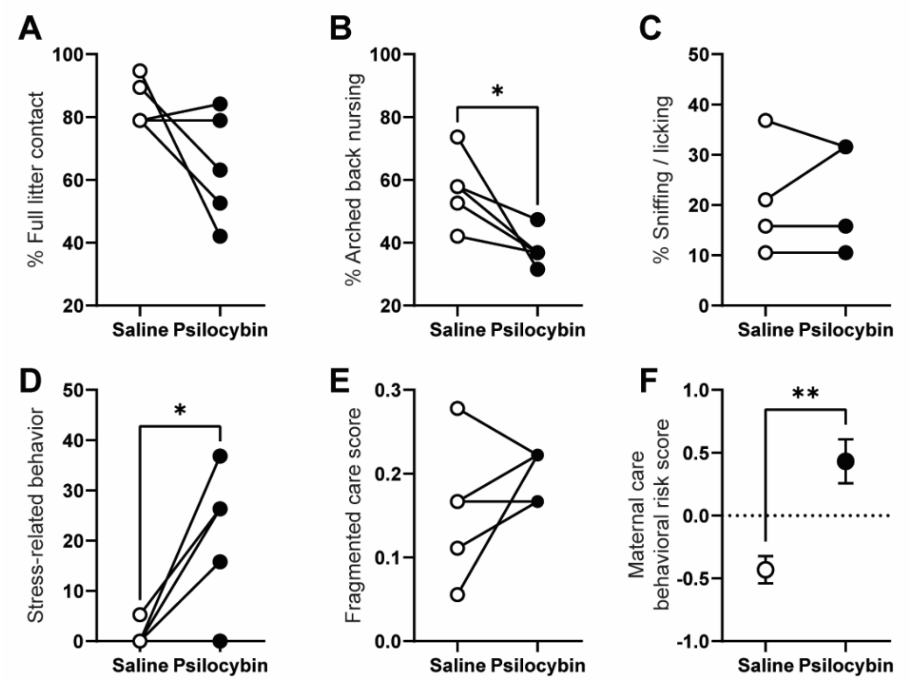

Data illustrated in **A–F** represent maternal behaviors exhibited by dams (n=5) exposed to saline on day one (PPD6) and psilocybin on day two (PPD7), measured during a 1 h observation immediately following each injection. Percentage of observations made (**A**) in contact with the entire litter, (**B**) engaging in arched back nursing, (**C**) sniffing or licking their pups, (**D**) exhibiting stress-related behaviors. (**E**) Fragmented care score indicating behavioral switching between pup-directed and non-pup-directed behaviors. (**F**) Maternal care behavioral risk score, an integrative measure of global maternal care impairments calculated by averaging z-normalized results from independent maternal care behaviors (arched back nursing, sniffing / licking, stress-related behaviors and fragmented care), such that the greater the risk score, the greater the global impairment. Results are presented as before-and-after plots and statistical comparisons between groups are made using a paired t test. *$p < 0.05$, **$p < 0.01$. Exact P values are provided in the Source Data.

Supplementary Fig. 5: Postpartum females show reduced expression of serotonergic system-related mRNA transcripts

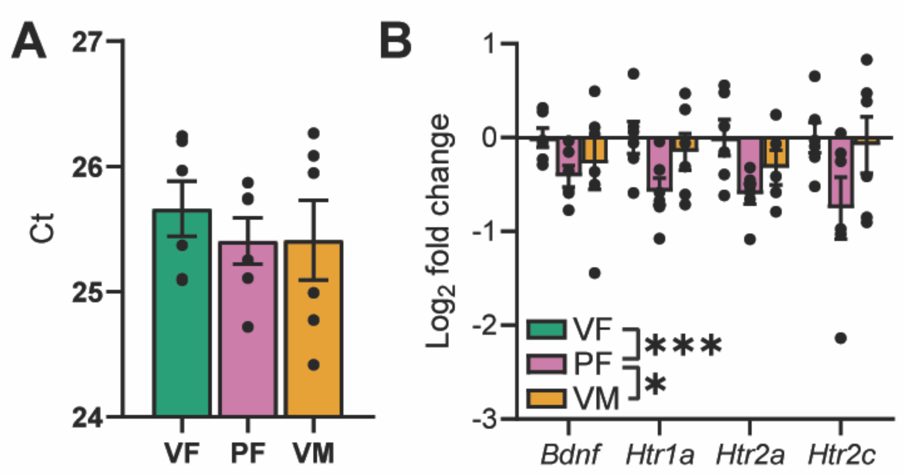

(**A**) Ct values across reproductive state for the housekeeping control gene, beta-2 microglobulin. (**B**) Relative abundance ($\log_2$ fold change) of mRNA transcripts encoding various proteins in virgin female (VF), postpartum female (PF), and virgin male (VM) mice. Individual data in **A** are presented as aligned dot plots and as bar charts representing mean ± SEM. Data in **B** are presented as means – SEM. Data were analyzed with a one-way ANOVA and significant ANOVA in **B** followed by planned comparisons between postpartum females and all other groups. N=6 mice/group *$p < 0.05$, ***$p < 0.001$. Exact P values are provided in the Source Data.

Supplementary Table 1. Pup weights and numbers

Group	Mean ± SEM Number of Pups	Mean ± SEM Pup Weight (g) on PPD 4	Mean ± SEM Pup Weight (g) on PPD 8
Control Saline	6 ± 1	2.49 ± 0.13	4.49 ± 0.19
Control Psilocybin	7 ± 1	2.89 ± 0.25	4.88 ± 0.32
Stress Saline	6 ± 1	2.82 ± 0.16	4.94 ± 0.18
Stress Psilocybin	6 ± 0.4	2.64 ± 0.14	4.74 ± 0.17

Supplementary Table 2. Parameters of MS/MS analysis for target analytes

Analytes	Precursor	Product Ion	DP (volts)	CE (volts)	CXP (volts)
**Psilocybin**	285	205	126	23	22
		240	126	25	28
**Psilocin**	205	160	46	21	20
		58	46	39	24
**Bufotenine-D4**	209	164	36	21	10
		60	36	15	8

Abbreviations: DP = declustering potential, CE = collision energy, CXP = collision exit potential

Supplementary Data 1. Maternal behavior ethogram and statistical summary

*p < 0.05, **p < 0.01, ***p < 0.001 and ****p < 0.0001. Exact P values are provided in the Source Data.
