# Supplementary material for: Psilocybin during the postpartum period induces long-lasting adverse effects in both mothers and offspring
Source: Nat Commun. 2025 Sep 30;16:8630. doi: 10.1038/s41467-025-64371-5 (PMC12485072; doi:10.1038/s41467-025-64371-5)
Supplement: Supplementary file 2 — Description of Additional Supplementary Files [file 41467_2025_64371_MOESM2_ESM.pdf]

Description of Additional Supplementary Files

Supplementary Data 1: Maternal behavior ethogram and statistical summary.
*$p < 0.05$, **$p < 0.01$, ***$p < 0.001$ and ****$p < 0.0001$. Exact P values are provided in the Source Data.

Supplementary Movie 1: Video example of maternal stress behavior.
