# Supplementary material for: Psilocybin during the postpartum period induces long-lasting adverse effects in both mothers and offspring
Source: Nat Commun. 2025 Sep 30;16:8630. doi: 10.1038/s41467-025-64371-5 (PMC12485072; doi:10.1038/s41467-025-64371-5)
Supplement: Supplementary file 5 — Reporting Summary [file 41467_2025_64371_MOESM5_ESM.pdf]

nature portfolio

Corresponding author(s): Danielle S. Stolzenberg, David E. Olson

Last updated by author(s): Aug 20, 2025

Reporting Summary

Nature Portfolio wishes to improve the reproducibility of the work that we publish. This form provides structure for consistency and transparency in reporting. For further information on Nature Portfolio policies, see our Editorial Policies and the Editorial Policy Checklist.

Statistics

For all statistical analyses, confirm that the following items are present in the figure legend, table legend, main text, or Methods section.

n/a	Confirmed	
☐	☒	The exact sample size (*n*) for each experimental group/condition, given as a discrete number and unit of measurement
☐	☒	A statement on whether measurements were taken from distinct samples or whether the same sample was measured repeatedly
☐	☒	The statistical test(s) used AND whether they are one- or two-sided<br>*Only common tests should be described solely by name; describe more complex techniques in the Methods section.*
☒	☐	A description of all covariates tested
☐	☒	A description of any assumptions or corrections, such as tests of normality and adjustment for multiple comparisons
☐	☒	A full description of the statistical parameters including central tendency (e.g. means) or other basic estimates (e.g. regression coefficient) AND variation (e.g. standard deviation) or associated estimates of uncertainty (e.g. confidence intervals)
☐	☒	For null hypothesis testing, the test statistic (e.g. *F*, *t*, *r*) with confidence intervals, effect sizes, degrees of freedom and *P* value noted<br>*Give P values as exact values whenever suitable.*
☒	☐	For Bayesian analysis, information on the choice of priors and Markov chain Monte Carlo settings
☒	☐	For hierarchical and complex designs, identification of the appropriate level for tests and full reporting of outcomes
☐	☒	Estimates of effect sizes (e.g. Cohen's *d*, Pearson's *r*), indicating how they were calculated

Our web collection on statistics for biologists contains articles on many of the points above.

Software and code

Policy information about availability of computer code

Data collection	QuantStudioTM 7 Pro
Data analysis	ANY-maze software version 7.43, Noldus Observer XT 12.5, GraphPad Prism v10.5, QuatStudioTM Design and Analysis Software 2.8.0, SCIEX MultiQuant 3.0

For manuscripts utilizing custom algorithms or software that are central to the research but not yet described in published literature, software must be made available to editors and reviewers. We strongly encourage code deposition in a community repository (e.g. GitHub). See the Nature Portfolio guidelines for submitting code & software for further information.

Data

Policy information about availability of data

All manuscripts must include a data availability statement. This statement should provide the following information, where applicable:

- Accession codes, unique identifiers, or web links for publicly available datasets
- A description of any restrictions on data availability
- For clinical datasets or third party data, please ensure that the statement adheres to our policy

The raw data for this manuscript is available on Figshare (https://figshare.com/s/5667219ba4c47adbbb3b).

Research involving human participants, their data, or biological material

Policy information about studies with human participants or human data. See also policy information about sex, gender (identity/presentation), and sexual orientation and race, ethnicity and racism.

Reporting on sex and gender	*Use the terms sex (biological attribute) and gender (shaped by social and cultural circumstances) carefully in order to avoid confusing both terms. Indicate if findings apply to only one sex or gender; describe whether sex and gender were considered in study design; whether sex and/or gender was determined based on self-reporting or assigned and methods used. Provide in the source data disaggregated sex and gender data, where this information has been collected, and if consent has been obtained for sharing of individual-level data; provide overall numbers in this Reporting Summary. Please state if this information has not been collected. Report sex- and gender-based analyses where performed, justify reasons for lack of sex- and gender-based analysis.*
Reporting on race, ethnicity, or other socially relevant groupings	*Please specify the socially constructed or socially relevant categorization variable(s) used in your manuscript and explain why they were used. Please note that such variables should not be used as proxies for other socially constructed/relevant variables (for example, race or ethnicity should not be used as a proxy for socioeconomic status). Provide clear definitions of the relevant terms used, how they were provided (by the participants/respondents, the researchers, or third parties), and the method(s) used to classify people into the different categories (e.g. self-report, census or administrative data, social media data, etc.) Please provide details about how you controlled for confounding variables in your analyses.*
Population characteristics	*Describe the covariate-relevant population characteristics of the human research participants (e.g. age, genotypic information, past and current diagnosis and treatment categories). If you filled out the behavioural & social sciences study design questions and have nothing to add here, write "See above."*
Recruitment	*Describe how participants were recruited. Outline any potential self-selection bias or other biases that may be present and how these are likely to impact results.*
Ethics oversight	*Identify the organization(s) that approved the study protocol.*

Note that full information on the approval of the study protocol must also be provided in the manuscript.

Field-specific reporting

Please select the one below that is the best fit for your research. If you are not sure, read the appropriate sections before making your selection.

☒ Life sciences ☐ Behavioural & social sciences ☐ Ecological, evolutionary & environmental sciences

For a reference copy of the document with all sections, see nature.com/documents/nr-reporting-summary-flat.pdf

Life sciences study design

All studies must disclose on these points even when the disclosure is negative.

Sample size	For behavior analyses we calculated a power analysis based on the maximum number of required groups = 8 (2 stimulus conditions x 2 drug conditions x 2 sexes). Power analyses (G*Power 3.1 software, Germany) indicate that 9 animals per group would be sufficient to detect statistical significance (Effect size f= 0.33, a error probability – 0.5, power = 0.8). Therefore, we aimed for 9 male and 9 female offspring per group. To achieve this sample size, we aimed for 11 dams per group (to account for attrition).
Data exclusions	Prior to conducting any statistical analyses, statistical outliers were determined by a ROUT test with Q = 1% and removed. For novel object recognition data, mice that did not meet investigation criteria were removed. The only other data that were excluded from analyses, were due to experimenter error (e.g. problems during video acquisition, students loosing videos, or sucrose bottles leaking). For the post-wean test battery in dams, 15 videos were excluded out of a total of 245 videos. For the offspring test battery, 24 videos were excluded out of a total of 510 videos. For the virgin female test battery, 1 video of out 90 was excluded. For the offspring directly exposed to psilocin, video recordings of the forced swim test malfunctioned for 3 mice. For the head twitch response experiment comparing virgin and postpartum mice with or without ketanserin pre-treatment, 2 videos were cut short and therefore excluded from analysis. Excluded data are indicated where applicable in the Source data file.
Replication	Data illustrated in figures 1-3 represent four independent cohorts of female mice were tested for maternal behavior by 5 independent experimenters over 4 months. Data illustrated in figure 4 illustrate offspring who were tested in 7 independent cohorts by 8 experimenters over 5 months. Data illustrated in figures 6-7 were collected in an entirely new colony of mice in a different animal facility following a lab relocation and tested by more than 2 independent experimenters over 6 months.
Randomization	With respect to stress and control groups, roughly equal numbers of animals were tested within each cohort. As pregnancies were not timed and mice gave birth on different dates, group assignment was pseudo-randomized to balance the treatment-assignments on a given day. Otherwise, animals were randomly assigned to cohorts with numbers of animals assigned to each group. For the comparison between virgin and postpartum mice, each virgin mouse was yoked to the testing schedule of a postpartum mouse. Equal numbers of mice assigned to each drug treatment group were tested in each cohort (total of 3). For direct psilocin experiments, male and female offspring in each litter were treated with vehicle or psilocin such that control animals were littermates. No more than 2 females and 2 males (1 per group) were used from each litter.

Blinding | During all manual behavior testing and coding, all experimenters were blind to drug treatment conditions throughout all experiments. During all manual coding, raters were trained to a high level of agreement (>90%). Blinding was not possible for stress condition during maternal behavior coding as mice were housed in different vivarium rooms and experimenters that administered the stress or mock treatment also recorded behavioral data. All analyses were completed prior to revealing treatment group codes.

Reporting for specific materials, systems and methods

We require information from authors about some types of materials, experimental systems and methods used in many studies. Here, indicate whether each material, system or method listed is relevant to your study. If you are not sure if a list item applies to your research, read the appropriate section before selecting a response.

Materials & experimental systems

n/a	Involved in the study
☒	☐ Antibodies
☒	☐ Eukaryotic cell lines
☒	☐ Palaeontology and archaeology
☐	☒ Animals and other organisms
☒	☐ Clinical data
☒	☐ Dual use research of concern
☒	☐ Plants

Methods

n/a	Involved in the study
☒	☐ ChIP-seq
☒	☐ Flow cytometry
☒	☐ MRI-based neuroimaging

Animals and other research organisms

Policy information about studies involving animals; ARRIVE guidelines recommended for reporting animal research, and Sex and Gender in Research

Laboratory animals | Adult virgin female and male mus musculus (mice) were obtained from Jackson Laboratories (Stock # 000664) at six weeks of age and bred in our laboratory to generate postpartum mice. Offspring were weaned at 21 days and tested at 87-150 days of age. Male intruders were also C57BL6/J but derived from breeders in our laboratory. Virgin females and dams used to test the acute effects of psilocybin were bred in our breeding colony. Animals used in figures 6-7 were unrelated to animals used in figures 1-5. These C57BL6/J mice were also obtained from Jax once the new laboratory was set up.

Wild animals | N/A

Reporting on sex | Offspring of both sexes were tested in all experiments. Sex was considered in the experimental design and incorporated into the power analysis. All data were tested for sex effects and disaggregated by sex.

Field-collected samples | N/A

Ethics oversight | All procedures were conducted in compliance with the University of California, Davis Institutional Animal Care and Use Committee.

Note that full information on the approval of the study protocol must also be provided in the manuscript.

Plants

Seed stocks | *Report on the source of all seed stocks or other plant material used. If applicable, state the seed stock centre and catalogue number. If plant specimens were collected from the field, describe the collection location, date and sampling procedures.*

Novel plant genotypes | *Describe the methods by which all novel plant genotypes were produced. This includes those generated by transgenic approaches, gene editing, chemical/radiation-based mutagenesis and hybridization. For transgenic lines, describe the transformation method, the number of independent lines analyzed and the generation upon which experiments were performed. For gene-edited lines, describe the editor used, the endogenous sequence targeted for editing, the targeting guide RNA sequence (if applicable) and how the editor was applied.*

Authentication | *Describe any authentication procedures for each seed stock used or novel genotype generated. Describe any experiments used to assess the effect of a mutation and, where applicable, how potential secondary effects (e.g. second site T-DNA insertions, mosiacism, off-target gene editing) were examined.*
